# Statins induce insulin-degrading enzyme secretion from astrocytes via an autophagy-based unconventional secretory pathway

**DOI:** 10.1186/s13024-015-0054-3

**Published:** 2015-10-31

**Authors:** Sung Min Son, Seokjo Kang, Heesun Choi, Inhee Mook-Jung

**Affiliations:** Department of Biochemistry & Biomedical Sciences, Seoul National University College of Medicine, 103 Daehak-ro, Jongro-gu, Seoul 110-799 Korea; Neuroscience Research Institute, Seoul National University College of Medicine, Seoul, Korea

**Keywords:** Statin, Insulin-degrading enzyme (IDE), Autophagy-based unconventional secretion, Amyloid-β (Aβ), Alzheimer’s disease (AD)

## Abstract

**Background:**

Insulin degrading enzyme (IDE) is a major protease of amyloid beta peptide (Aβ), a prominent toxic protein in Alzheimer’s disease (AD) pathogenesis. Previous studies suggested that statins promote IDE secretion; however, the underlying mechanism is unknown, as IDE has no signal sequence.

**Results:**

In this study, we found that simvastatin (0.2 μM for 12 h) induced the degradation of extracellular Aβ_40_, which depended on IDE secretion from primary astrocytes. In addition, simvastatin increased IDE secretion from astrocytes in a time- and dose-dependent manner. Moreover, simvastatin-mediated IDE secretion was mediated by an autophagy-based unconventional secretory pathway, and autophagic flux regulated simvastatin-mediated IDE secretion. Finally, simvastatin activated autophagy via the LKB1-AMPK-mTOR signaling pathway in astrocytes.

**Conclusions:**

These results demonstrate a novel pathway for statin-mediated IDE secretion from astrocytes. Modulation of this pathway could provide a potential therapeutic target for treatment of Aβ pathology by enhancing extracellular clearance of Aβ.

**Electronic supplementary material:**

The online version of this article (doi:10.1186/s13024-015-0054-3) contains supplementary material, which is available to authorized users.

## Background

Alzheimer’s disease (AD) is the most common form of dementia; it is characterized by senile plaques, neurofibrillary tangles, and neuronal cell death [1, 2]. Abnormally increased levels of amyloid beta peptides (Aβ) lead to formation of extracellular senile plaques and are associated with neurodegeneration in AD [3, 4]. The Aβ levels in the brain are not only determined by the rate of production by amyloid precursor protein (APP) processing [5, 6], but also by several clearance mechanisms. These include proteolytic degradation of extracellular Aβ by cell surface-localized and/or secreted proteases such as neprilysin (NEP), matrix metalloproteinase-9 (MMP-9), and insulin-degrading enzyme (IDE) [7–9]. NEP is located mainly in the plasma membrane, and its catalytic domain faces the extracellular space [7]. MMP-9 and IDE can be secreted extracellularly and degrade extracellular Aβ, despite the fact that IDE has no signal sequence for secretion through a conventional secretory pathway [10]. Many studies have demonstrated that IDE is secreted [11]; however, the mechanism of secretion is still elusive.

Macroautophagy (hereafter referred to as autophagy) is a fundamental biological process in eukaryotes and has an impact on essential biological processes including aging, cancer, neurodegenerative diseases, and metabolic disorders [12, 13]. Autophagy is currently best known as a degradative pathway that delivers cytoplasmic materials and organelles to the lysosomes for degradation [14]. All autophagy-related processes include the formation of double-membrane structures called autophagosomes and are induced by the inhibition of the mammalian target of rapamycin (mTOR) signaling pathway. Autophagosomes and their contents undergo clearance upon fusion with an endosome (amphisomes) or lysosome (autolysosomes) for degradation and recycling (autophagic flux) [12, 13, 15]. However, recent studies show that autophagy also has a role in non-autophagic processes, especially in the secretory pathway [16]. In eukaryotic cells, the autophagy-based secretory pathway regulates the unconventional secretion of several cytosolic proteins or factors such as IL (interleukin)-1β, IL-18, High-mobility Group Box 1 (HMGB1), ATP (adenosine triphosphate), Aβ, and von Willebrand factor [17–20]. These proteins share important features, including the lack of a signal sequence for conventional secretion, and the contribution of autophagy-related (Atg) protein to their secretion.

Several studies report that increased cholesterol levels might be related to AD [21, 22], and that statin-mediated inhibition of 3-hydroxy-3-methylglutaryl-coenzyme A (HMG-CoA) reductase decreases cholesterol levels; thus, reducing Aβ levels [23, 24]. However, this is controversial [22]. Several studies have demonstrated that statins can decrease the generation of Aβ by enhancing non-amyloidogenic processing of APP [25, 26]. In addition, statins also impair the generation of isoprenoids, which play important roles in the post-translational modification of proteins in the Rho and Rab families [26, 27]. Isoprenoids regulate the localization and biological function of Rho and Rab proteins, and affect Aβ generation by modulating APP processing [27]. Previous studies have also shown that statins promote Aβ degradation by microglia via IDE secretion [24]. However, the molecular mechanisms by which statins could offer protection against AD have not been studied extensively.

In this study, we found that IDE secretion from primary astrocytes was increased by statins in a time- and concentration-dependent manner, and statin-induced IDE secretion was associated with autophagy-based unconventional secretion. Additionally, we found that autophagic flux is important in IDE secretion and that statin activates autophagy in astrocytes via the LKB1-AMPK-mTOR signal pathway. These results indicate that IDE is secreted from astrocytes via an autophagy-based secretory pathway, and that regulation of autophagy is a potential therapeutic target in Aβ pathology.

## Results

### Statins induce extracellular secretion of functional IDE from astrocytes

**Fig. 1:**
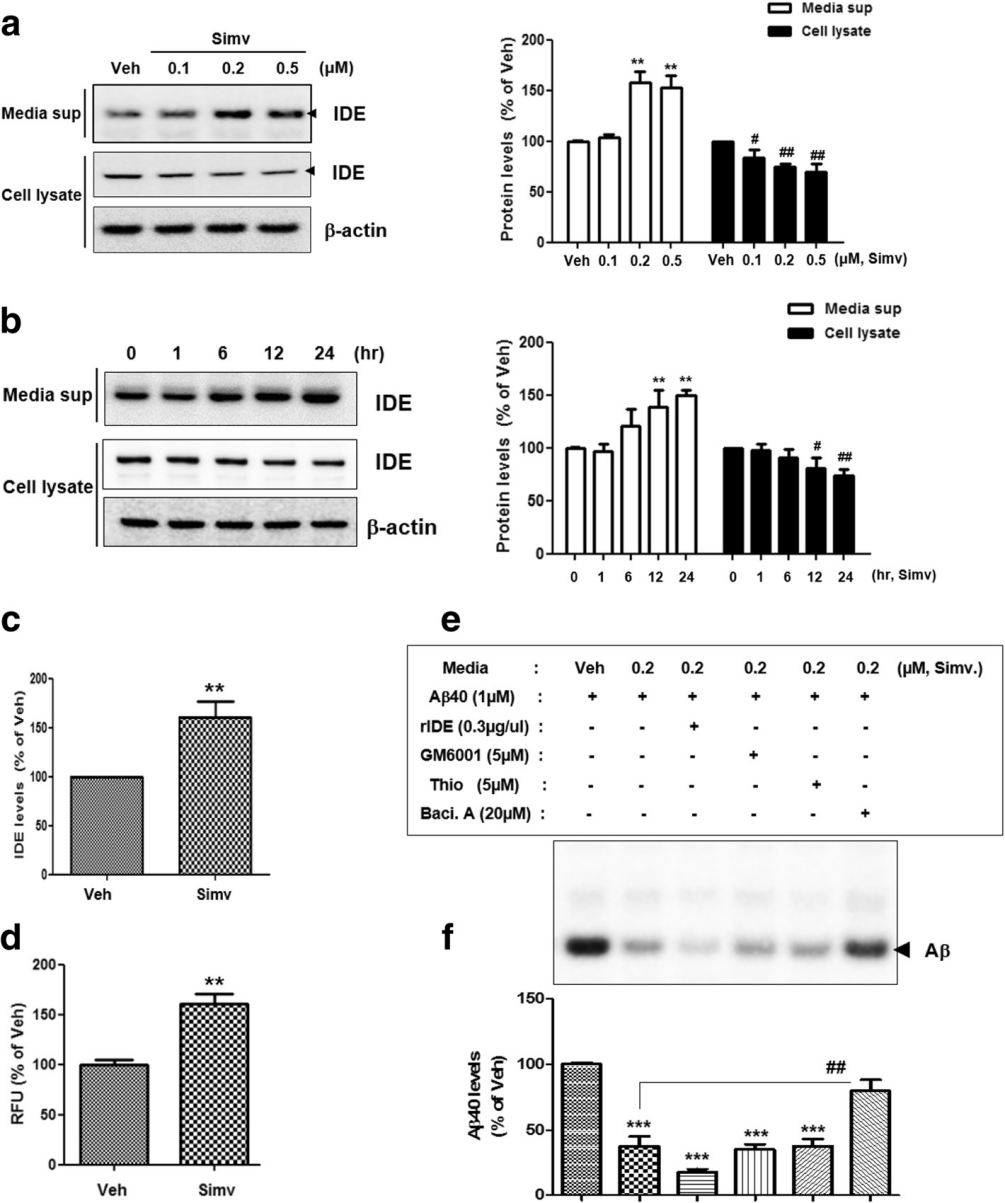
Statin treatment induces extracellular secretion of functional insulin-degrading enzyme (IDE) from astrocytes. **a & b** Increased IDE levels secreted from the primary astrocytes by simvastatin in a concentration-(**a**) and time-(**b**) dependent manner. The time points mean the time period of simvastatin treatment. Blots are representative of at least three independent experiments (*N* = 3 experiments). ** *p* < 0.01, # *p* < 0.05, ## *p* < 0.01 vs. vehicle-treated cells. **c** Secreted IDE levels by simvastatin with IDE ELISA. **d** IDE enzymatic activity in the media from simvastatin-treated cells. (**e & f**) Cell-free Aβ degradation assay. The arrowhead indicates remaining Aβ_40_ levels. Data were obtained from at least three replicates for each group (*N* = 3 experiments). rIDE, recombinant IDE protein; Thio, thiorphan; Baci. A, Bacitracin A. ** *p* < 0.01, *** *p* < 0.001 vs. vehicle-treated cells; ## *p* < 0.01 vs. cells treated with simvastatin

Previous studies have shown that astrocytes are the main source for IDE in AD pathology [28]. Therefore, we first determined whether statins regulate IDE levels in the extracellular space of astrocytes. We found that simvastatin increased IDE secretion from primary astrocytes in a time- and dose-dependent manner (Fig. 1a-c), and that IDE levels in the cells were reduced conversely (Fig. 1a, b). Because statins are known to lower cholesterol levels, we checked whether statins regulate cholesterol levels in astrocytes. By using a filipin assay, we found that simvastatin reduced intracellular cholesterol levels (Additional file 1: Figure S1A,B). To determine whether only simvastatin induced IDE secretion from astrocytes, one of the other known statins, fluvastatin, was applied to astrocytes; we found that fluvastatin also increased IDE secretion (Additional file 2: Figure S2A,B). To examine whether the IDE secreted by statin treatment functions as insulysin (having an insulin-degrading function), we utilized an IDE enzymatic activity assay. We detected an increased in fluorescent intensity generated by the cleavage of fluorometric IDE substrates in the statin-treated astrocyte-conditioned media (ACM) (Fig. 1d). In an alternative approach, we performed an Aβ degradation assay. When the statin-treated ACM was incubated with the recombinant Aβ_40_ peptide, the level of the remaining Aβ_40_ peptide was reduced (Fig. 1e; lane 1 vs. lane 2). Furthermore, the reduced Aβ_40_ levels after incubation with the statin-treated ACM were restored when bacitracin A, an IDE inhibitor, was added (Fig. 1e; lane 2 vs. lane 6). The inhibitors of other known Aβ degrading enzymes including thiorphan, a NEP inhibitor, or GM6001, a MMP inhibitor, did not restore the reduced Aβ_40_ levels by the statin. These data demonstrate that statins induce IDE secretion from astrocytes, and statin-induced IDE functions as a protease to degrade the Aβ peptide.

### Statin-mediated IDE secretion from astrocytes is associated with an autophagy-based unconventional secretory pathway

**Fig. 2:**
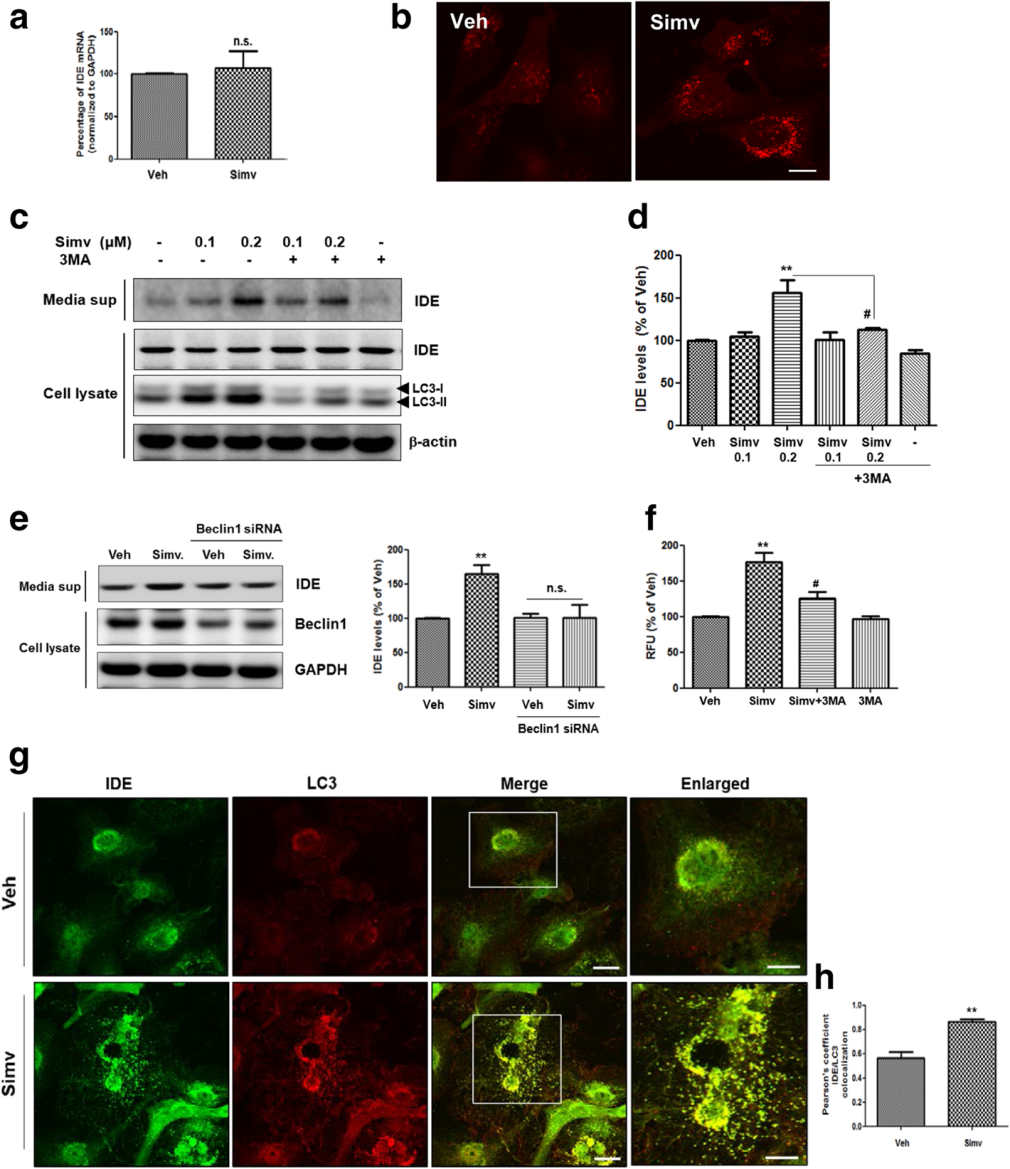
Statin − mediated IDE secretion from astrocytes is associated with an autophagy-based unconventional secretory pathway. **a** Quantification of *ide* mRNA levels by qPCR. **b** Autophagy activation in astrocytes by simvastatin. Anti-LC3 antibody was used to detect autophagy. Scale bar represents 5 μm. **c** Western blot analysis of secreted IDE levels from astrocytes after treatment with the autophagy inhibitor in the presence of simvastatin. **d** Quantitative analysis of Fig. 2c. Data were obtained from at least three replicates for each group (*N* = 3 experiments). **e** Western blot analysis of IDE levels in the media from Beclin1 knock-down astrocytes. **f** IDE enzymatic activity in the media from simvastatin and/or 3MA-treated cells. **g** Confocal microscopy images of IDE expression in the autophagosomes. Scale bar represents 8 and 6 μm (enlarged images). **h** Quantitative analysis of IDE in association with the autophagosome marker (LC3) with the Image J program (NIH, MD, USA). Data were obtained from at least three replicates (*N* = 3 experiments). ** *p* < 0.01 vs. vehicle-treated cells; # *p* < 0.05 vs. cells treated with simvastatin. n.s. indicates no significant difference

To examine the mechanism of statin-induced IDE secretion, transcript levels of IDE after statin treatment were measured by quantitative real-time PCR (qPCR). Simvastatin did not alter *ide* mRNA levels in astrocytes (Fig. 2a). Because IDE has no signal sequence for secretion, we further investigated the secretory mechanism of IDE. Previous studies showed that statins could stimulate secretion of IDE proteins via an unconventional pathway in association with exosomes [24]. To determine whether statin-induced IDE secretion is associated with exosomes in astrocytes, both exosomes and non-exosome fractions were isolated from vehicle- or statin-treated ACM. We found that simvastatin increased secreted IDE levels both in the exosomes and non-exosome fractions (Additional file 3: Figure S3), indicating that the secretory pathway for IDE is mediated by both exosome- and non-exosome-associated pathways. To determine the exact mechanisms of IDE secretion by statin, we focused on the autophagy-based unconventional secretory pathway. We used immunostaining and western blot analysis and found that statin treatment activated autophagy (Fig. 2b,c). In addition, we found that statin-induced IDE secretion was blocked by autophagy inhibition (Fig. 2c–e). Treatment with 3-methyladenine (3MA), a well-known autophagy inhibitor (Fig. 2c,d), or the knock-down of Beclin1, a key component of autophagy-initiation complex, reduced statin-induced IDE secretion from astrocytes (Fig. 2e). In the IDE enzymatic assay, 3MA inhibited increased IDE activity by statin treatment (Fig. 2f). To visualize whether IDE exists in the autophagosomes, IDE and LC3 were stained with their specific antibodies, followed by observation using confocal microscopy. Immunocytochemistry data showed that IDE was located in the autophagosomes, and simvastatin increased the level of IDE in the autophagosomes (Fig. 2g,h). Taken together, these data imply that statin-induced IDE secretion is tightly regulated by the autophagy pathway in astrocytes.

### Statin-mediated IDE secretion from astrocytes is regulated by autophagic flux

**Fig. 3:**
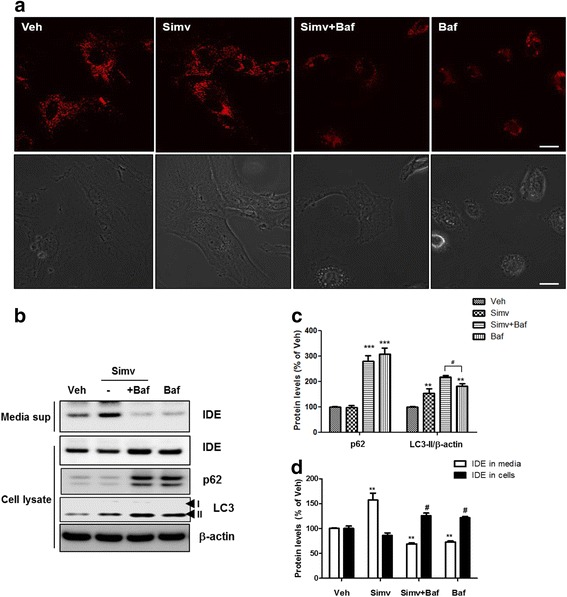
Statin-mediated IDE secretion from astrocytes is regulated by autophagic flux. **a** Lysosomal activity as determined with LysoTracker probe. Scale bar represents 10 μm. **b** Change in secreted IDE levels by inhibiting lysosome with bafilomycin. **c & d** Quantitative analysis of Fig. 3b (*N* = 3 experiments). Baf, bafilomycin. ** *p* < 0.01, # *p* < 0.05 vs. vehicle-treated cells

Previous studies showed that secretion of IL-1β, one of the substrates for autophagy-based unconventional secretion, was inhibited by lysosomal dysfunction that was induced by treatment with bafilomycin [17]. To further examine the statin-induced IDE secretory pathway, we determined if autophagic flux was blocked by bafilomycin, a lysosomotropic agent that prevents lysosomal acidification and autophagosomal cargo degradation. Using the LysoTracker probe, we found that bafilomycin inhibited lysosomal activity (Fig. 3a). In addition, treatment with bafilomycin decreased IDE secretion from astrocytes and led to accumulation of IDE proteins in the cells (Fig. 3b,c), suggesting that autophagic flux is important for IDE secretion.

### Statin activates autophagy via the AMPK-mTOR signaling pathway in astrocytes

**Fig. 4:**
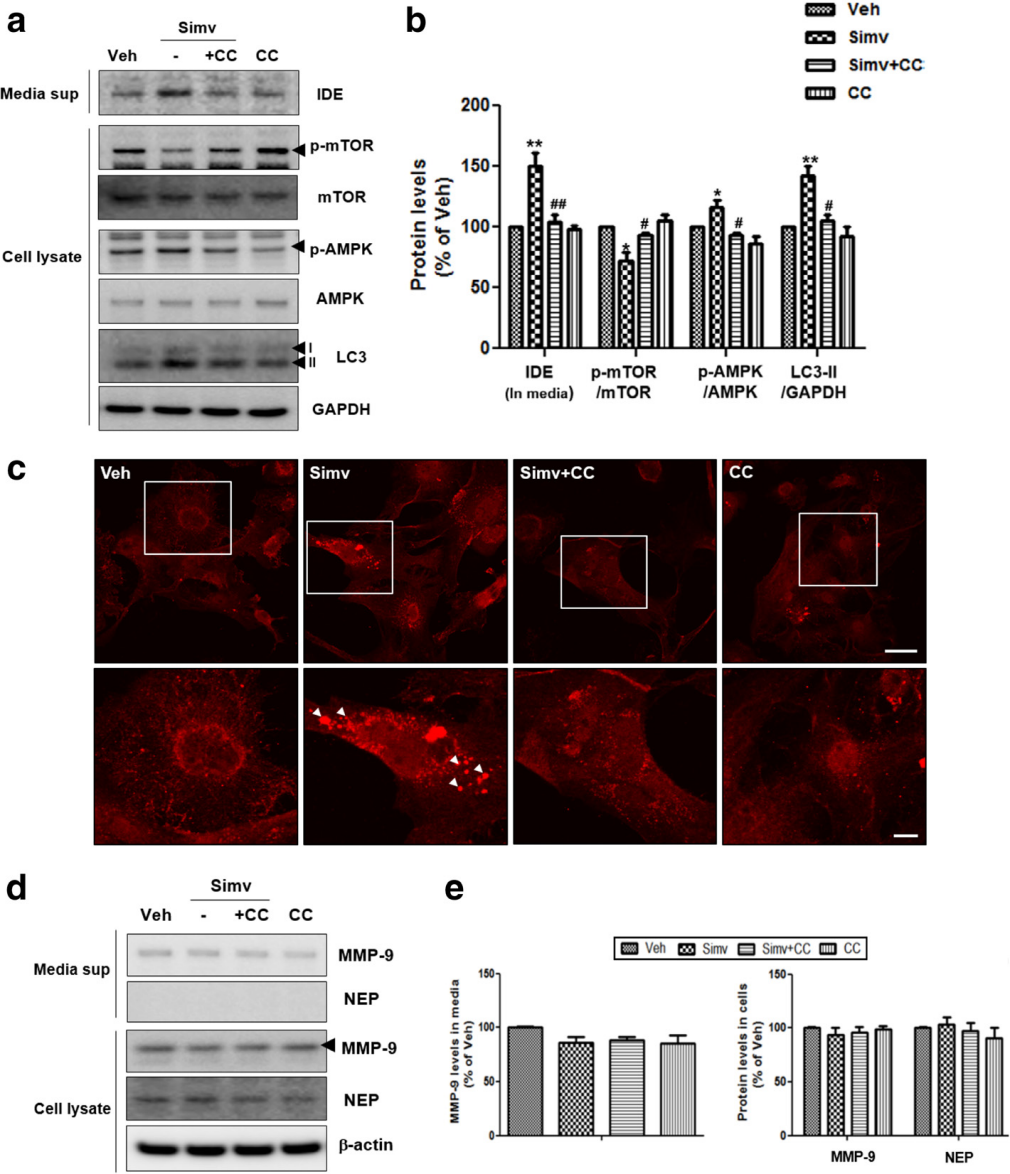
Statin activates autophagy via AMPK-mTOR signaling pathway in astrocytes. **a** Inhibition of statin-mediated autophagy activation and IDE secretion by treatment with AMPK inhibitor (Compound C; CC). **b** Quantitative analysis of Fig. 4a (*N* = 3 experiments). * *p* < 0.05, ** *p* < 0.01 vs. vehicle-treated cells; # *p* < 0.05, ## *p* < 0.01 vs. cells treated with simvastatin. **c** Inhibition of statin-mediated autophagy activation by treatment with compound C. Anti-LC3 antibody was used to detect autophagy. Scale bar represents 6 μm. Lower panels show figures under higher magnification. Scale bar represents 2 μm. **d** The effect of statins on regulation of MMP-9 and NEP levels. **e** Quantitative analysis of Fig. 4d (*N* = 3 experiments)

To investigate the mechanism of autophagy activation by statins, astrocytes were treated with an inhibitor of AMP-activated protein kinase (AMPK). Previous studies have shown that statin treatment induces AMPK activation [29] and the AMPK-mTOR signaling pathway regulates autophagy initiation [30]. When compound C, a well-known AMPK-specific inhibitor [31], was applied with simvastatin to astrocytes, it blocked the statin-induced phosphorylation of AMPK (Fig. 4a,b). We also observed that statin treatment decreased the phosphorylation of mTOR, and increased LC3-II levels (Fig. 4a, b). Conversely, compound C reversed these changes in mTOR kinase and LC3-II levels by statins (Fig. 4a, b). In addition, compound C inhibited statin-induced IDE secretion significantly (Fig. 4a, b). To visualize the alterations in autophagosome formation caused by the AMPK-specific inhibitor directly, immunocytochemistry was performed after co-treatment with compound C and statin. Statin treatment alone increased the punctate signal of LC3 (Fig. 4c), indicating enhanced autophagosome formation. Compound C prevented this increase, suggesting that AMPK signaling is required for statin treatment to promote autophagosome formation in astrocytes.

### Simvastatin does not regulate MMP-9 and NEP levels in astrocytes

In above data, simvastatin activated autophagy via AMPK-mTOR pathway, and statin-induced autophagy activation is neccessary for IDE secretion. To investigate whether other Aβ degradation enzymes such as MMP-9 and NEP can be also regulated by autophagy, MMP-9 and NEP levels were measured with media and cell lysates under simvastatin and/or Compound C treated condition. We found that simvastatin did not alter MMP-9 and NEP levels (Fig. 4d and e).

### LKB1, but not CaMKKβ, mediates the statin-mediated phosphorylation of AMPK

**Fig. 5:**
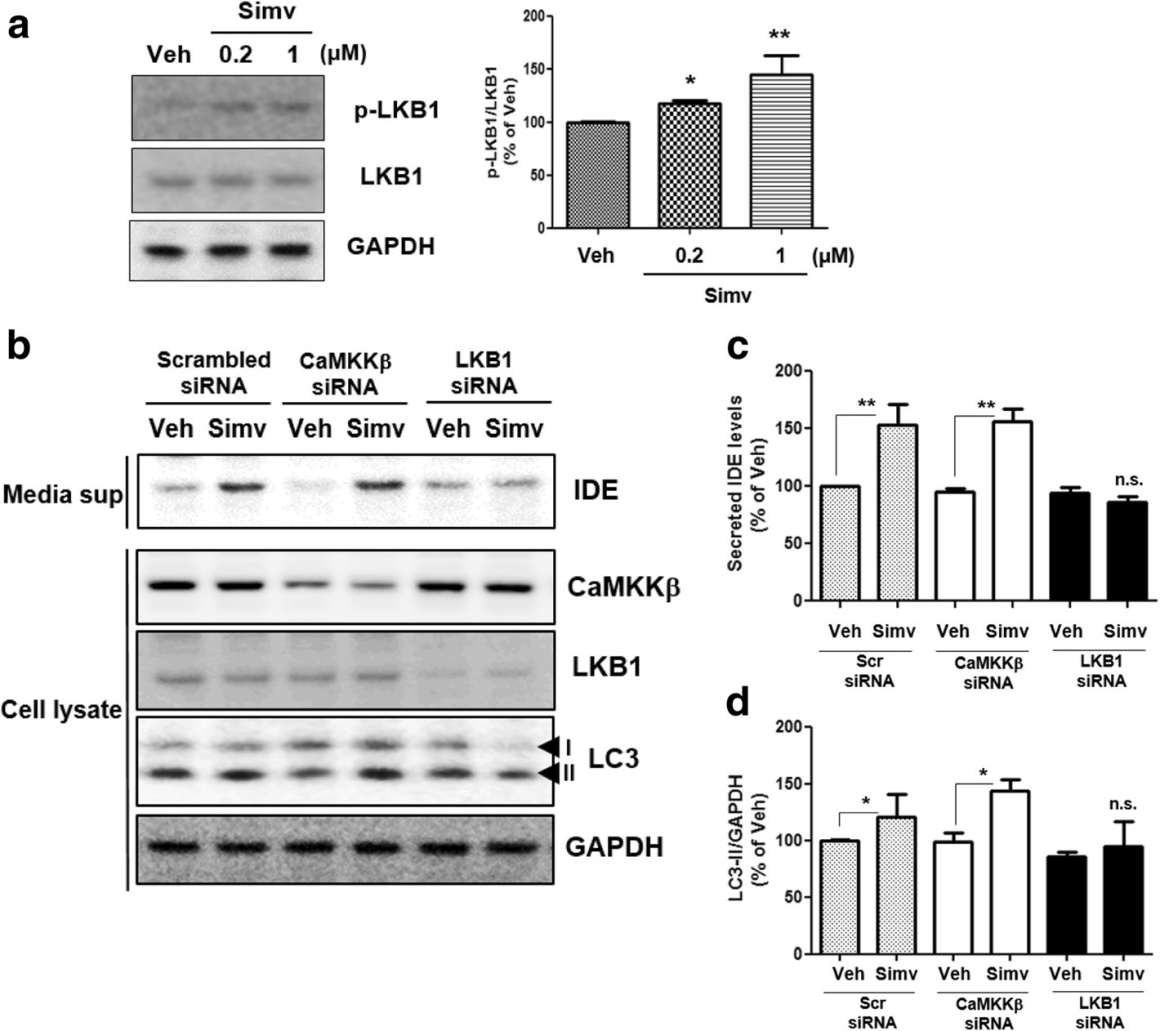
Statin-induced AMPK activation is mediated by LKB1 in astrocytes. **a** Increased phosphorylation of LKB1 by treatment with simvastatin. **b** Statin-induced autophagy activation and IDE secretion levels in CaMKKβ or LKB1 knock-downed cells. **c & d** Quantitative analysis of Fig. 5b (*N* = 3 experiments). * *p* < 0.05, ** *p* < 0.01 vs. vehicle-treated cells. n.s. indicates no significant difference

In previous studies, 2 upstream kinases were reported to activate AMPK, liver kinase B1 (LKB1) and Ca^2+^/calmodulin-dependent protein kinase kinase-beta (CaMKKβ) [32, 33]. We found that statin treatment induced LKB1 phosphorylation (Fig. 5a), and LKB1-specific siRNA failed to induce statin-induced phosphorylation of AMPK or generation of LC3-II (Fig. 5b-d), indicating that LKB1 is an AMPK upstream kinase for statin-induced autophagosome formation in astrocytes. When CaMKKβ-specific siRNA was transfected into astrocytes, statin-induced phosphorylation of AMPK and levels of LC3-II were not altered (Fig. 5b-d), indicating that CaMKKβ is not required for statin-induced autophagosome formation in astrocytes. These data imply that LKB1, not CaMKKβ, mediates statin-induced AMPK phosphorylation and then induces autophagosome formation.

## Discussion

**Fig. 6:**
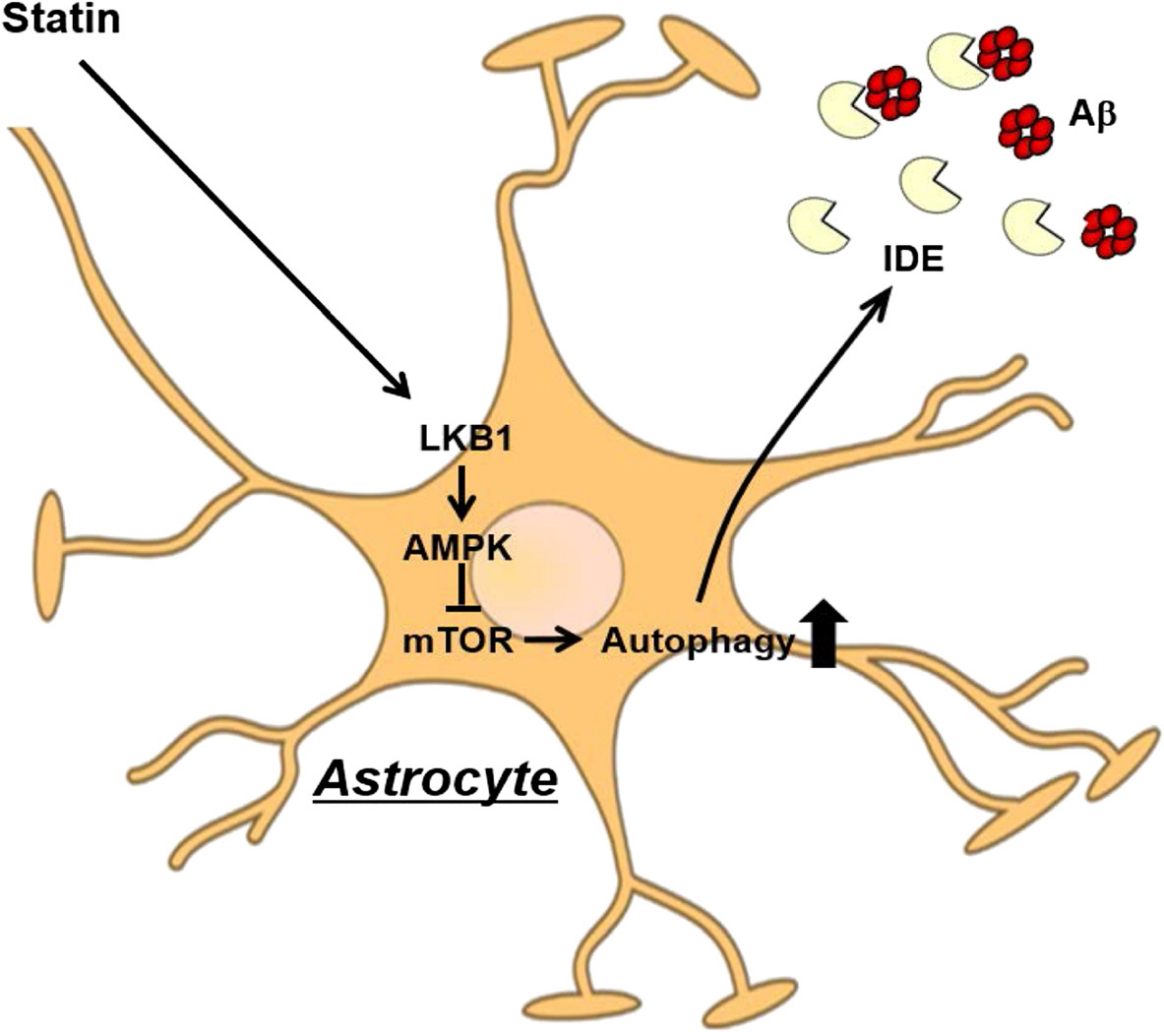
Schematic diagram. Statins activate autophagy via the LKB1-AMPK-mTOR signaling pathway in astrocytes, and statin-mediated IDE secretion is mediated by an autophagy-based unconventional secretory pathway. Finally, secreted IDE can significantly decrease in extracellular Aβ levels, thus, modulation of this pathway can regulate AD progression by enhancing Aβ clearance

In this study, we first demonstrated that statin treatment could induce extracellular IDE secretion from astrocytes via an autophagy-based unconventional secretory pathway (Fig. 6). Secreted IDE can significantly degrade extracellular Aβ peptides, indicating that it may be important for AD progression. In microglia, IDE, which has no known signal sequences, is secreted under statin-treated conditions via an exosome associated unconventional secretory pathway [24]. To determine whether statin-induced IDE secretion is associated with exosomes in astrocytes, both exosomes and non-exosome fractions were isolated from vehicle- or statin-treated astrocyte-conditioned media (ACM). We found that statin application increased IDE secretion from both the exosome and non-exosome fractions (Additional file 3: Figure S3), indicating that the secretory pathway for IDE is mediated by both exosome- and non-exosome-associated pathways. We also found that statin treatment increased functional IDE secretion in a time- and dose-dependent manner, and statin-induced IDE secretion was blocked by autophagy inhibition although IDE secretion at basal condition might be mediated by an autophagy-independent pathway (Fig. 2d-f). Because the fundamental role of autophagy is the clearance of protein aggregates and pathogens [13], we determined whether statin-mediated IDE secretion is regulated by the autophagy-lysosome pathway. When lysosomes were disrupted by the lysosomal inhibitor bafilomycin, IDE secretion was blocked, indicating that autophagic flux is important for statin-mediated IDE secretion. In this study, cell death was not observed under several drug-treated conditions (Additional file 4: Figure S4).

Astrocytes have an important role in maintaining neuronal homeostasis by providing energy and eliminating waste [34]. In addition, astrocytes are well positioned both metabolically and anatomically to play an important homeostatic role under basal conditions as well as pathological conditions. Previous studies have shown that at an early stage of AD, activated astrocytes surround and infiltrate Aβ plaque [35, 36]. Although their exact role in AD pathogenesis remains unknown, reactive astrocytes have been shown to play a role in the clearance of Aβ suggesting a neuroprotective role in AD [37]. For the removal of extracellular Aβ, astrocytes take up Aβ bound to membrane receptors, such as LRP1, via endocytosis and degrade Aβ [38]. In addition, astrocytes are capable of degrading Aβ enzymatically by secretion of MMP-9 and IDE extracellularly [39, 40]. Especially, in AD pathology, astrocytes are the main source for IDE [28]. Our results provide evidence that in statin-treated conditions, astrocytes can release IDE extracellularly via an autophagy-based secretory pathway, and the secreted IDE could then degrade extracellular Aβ. To determine whether secretion of other Aβ degrading enzymes such as NEP or MMP-9 is induced by autophagy similarly to IDE, we found that simvastatin did not induce secretion of NEP and MMP-9 (Fig. 4d and e). Previous studies have indicated that NEP is located mainly in the plasma membrane [7], and secretion of MMP-9 is mediated through a conventional secretory pathway, as MMP-9 has signal peptides for secretion [9]. Therefore, among Aβ-degrading enzymes, only IDE may be secreted by an autophagy-based secretory pathway.

There are many reports that elevated levels of cholesterol increase the risk for AD and that statins can regulate AD progression [21, 22]. Several studies have reported beneficial effects of statin treatment on AD pathology [24, 41]. However, some studies did not report any positive effects of statin; statin treatment actually increased Aβ levels [22, 42]. Such variable conclusions might stem from the fact that most of these studies were conducted with randomized concentrations of statins and time of statin treatment (early stage or late stage of AD) and the differential ability of statins to cross the blood-brain barrier (BBB) [24]. In our data, lysosomal dysfunction by treatment with bafilomycin inhibited statin-mediated IDE secretion. Because late-stage AD brains show autophagy accumulation and lysosomal dysfunction [43], it is possible that statins may not be beneficial when administered to patients with late-stage AD.

Abnormally pathological autophagic vacuoles (AVs) accumulate in the brains of patients with AD and in brains from animal models of AD [44]. The excessive autophagosome formation might result from increased induction via the AMPK-mTOR pathway and dysfunction in autophagy-lysosomal degradation [31, 43]. Because maintaining autophagic flux is important for cell survival [13], dysregulated autophagy could accelerate AD progression; however, the mechanism is not yet fully understood. In this study, impaired autophagic flux inhibited statin-mediated IDE secretion from astrocytes; the reduced IDE secretion from astrocytes could lead to an increase in extracellular Aβ levels. The regulation of Aβ levels is of great interest for AD treatment. Apart from the interference with Aβ generation, a promising alternative may be the enhancement of Aβ degradation by targeting Aβ-degrading enzymes [45]. Thus, regulating IDE secretion by statins in astrocytes may lead to new therapeutic approaches for sporadic AD.

## Conclusions

Our data collectively suggest that statin treatment can induce extracellular IDE secretion from astrocytes via an autophagy-based unconventional secretory pathway. Because secreted IDE can significantly decrease in extracellular Aβ levels, modulation of this pathway could provide a potential therapeutic target for treatment of Aβ pathology.

## Methods

### Cell cultures, drug treatments, and transfection

Primary astrocytes were prepared from newborn (P1) ICR mice as described previously [46]. The cells underwent two passages for the experiments and were grown in DMEM supplemented with 10 % fetal bovine serum (FBS; HyClone, Irvine, CA, USA) and 0.1 mg/ml P/S (Penicillin-Streptomycin; Sigma-Aldrich, St. Louis, MO, USA) at 37 °C in humidified 5 % CO_2_ incubator for 2 weeks. The cells were treated with several reagents alone, or co-treated with simvastatin followed by 24 h incubation. Reagents used in this study were thiorphan (T6031; NEP inhibitor), bacitracin A (B0125; IDE inhibitor), 3MA (M9281; autophagy blocker), bafilomycin (B1793; selective inhibitor of vacuolar-type H^+^-ATPase), simvastatin (S6196), fluvastatin (SML0038), STO-609 (S1318; CaMKKβ inhibitor), methyl-β-cyclodextrin (MβCD) (C4555; cholesterol‐lowering compound) from Sigma-Aldrich; compound C (171260; AMPK inhibitor) from Calbiochem (San Diego, CA, USA); GM6001 (142880-36-2; MMP inhibitor) from Tocris Bioscience (Bristol, UK). The siRNAs were purchased from Bioneer Inc. (Daejeon, Korea) (1330628 for *beclin 1*, 1432200 for *lkb1*, 1334167 for *camkk2*), and were transfected into the cells using RNAimax (13778) (Invitrogen, Carlsbad, CA) according to the manufacturer’s instructions.

### Western blot analysis

Harvested cell pellets and media were prepared as described previously [47]. Briefly, cell pellets were lysed in RIPA buffer (50 mM Tris-Cl, pH 8.0, 150 mM NaCl, 1 % NP-40, 0.5 % NaDoc, 0.1 % SDS) containing protease inhibitors (Sigma-Aldrich). After sonication at 4 °C, 10 μg of lysate was separated on SDS-PAGE gels and then transferred to PVDF membranes. Membranes were incubated with antibodies against the indicated proteins in this study. The antibodies for the western blot analysis were: anti-LC3B antibody (1:2,000; M152-3, MBL, Woburn, MA, USA; 2775, Cell Signaling Technology, Beverly, MA, USA); 6E10 (1:5000; SIG-39300, Covance, Princeton, NJ, USA); anti-p-LKB1 (S428), anti-AMPK (2532), anti-p-AMPK (T172) (2535) and anti-p-mTOR (S2448) (5536) (1:2000, Cell Signaling Technology); anti-LKB1 (sc-32245), anti-IDE (N-15) (sc-27265) and anti-mTOR (sc-1549) (1:1,000; Santa Cruz Biotechnologies Inc., Santa Cruz, CA, USA); anti-IDE (ab32216), anti-GAPDH (ab9485), anti-TSG101 (ab83) and anti-beclin1 (ab62472) (1:2,000; Abcam, Cambridge, MA, USA); anti-p62 (P0067), and anti-β-actin (A1978) (1:4,000; Sigma-Aldrich). Immunoreactivity was determined by chemiluminescence (GE Healthcare, Piscataway, NJ, USA). The chemiluminescence signal was detected with a digital image analyzer (LAS-3000; Fuji, Tokyo, Japan).

### Aβ degradation assay

The Aβ degradation assay was performed as previously described with modification [24]. Briefly, primary astrocytes grown in a 6-well plate were incubated with simvastatin or DMSO (vehicle) in serum-free DMEM for 24 h. The supernatant was then collected after centrifugation and incubated with 1 μM recombinant human Aβ_40_ (American Peptide, Sunnyvale, CA, USA) for 12 h at 37 °C in the absence or presence of 5 μM GM6001, 5 μM thiorphan, 20 μM bacitracin A or 0.3 μg/μl recombinant IDE (rIDE). Remaining Aβ_40_ levels were quantified with 6E10 antibody by western blot analysis.

### Trichloroacetic acid (TCA) precipitation

For analyzing protein in the medium, we performed TCA precipitation as previously described [48]. Briefly, cell medium was centrifuged at 2,400 g for 5 min to remove cell debris and then subjected to TCA precipitation (up to 10 %) (T6399, Sigma-Aldrich).

### IDE activity assay and IDE level measurement in the media

The IDE enzymatic activity in media was determined per manufacturer's protocol (CBA079, Calbiochem). Briefly, 50 μl of the concentrated media were loaded into in a 96-well plate, which contained an affinity-purified polyclonal antibody that recognizes IDE. The media were concentrated with an Amicon Ultra filter (UFC510024, Millipore, Billerica, MA). Following 1 h incubation, fluorometric IDE substrates were added and incubated for 2–4 h at 37 °C in the dark. The fluorescence was measured using an excitation wavelength of 320 nm and an emission wavelength of 405 nm. To determine the level of IDE in media, ELISA was performed per manufacturer’s protocol (MBS725082, Mybiosource, San Diego, CA, USA).

### Measurement of lysosomal activity

The measurement of lysosomal activity with LysoTracker-Red (L7528; Invitrogen) was performed as per manufacturer's protocol. The fluorescence intensity was observed using a confocal laser scanning microscope (FV10i-w, Olympus, Tokyo, Japan) and representative cells were selected and photographed.

### Quantitative real-time PCR (qRT-PCR)

To examine the levels of IDE mRNA, qRT-PCR was performed as previously described [49]. RNA was isolated using the RNeasyPlus Mini Kit (Qiagen, Valencia, CA, USA) and cDNA was generated using the RevertAid First Strand cDNA Synthesis Kit (Fermentas, Glen Burnie, MD). Real-time PCR was performed on the cDNA samples using ABI StepOne 2.1 (Applied Biosystems, Foster City, CA), and the following sense and antisense primers were used: 5′-CCGGCCATCCAGAG AATAGAA-3′ (sense), 5′- ACGGTATTCCCGTTTGTCTTCA-3′ (antisense) for IDE.

### Isolation and characterization of exosomes

Exosomes and non-exosome fractions in the media from astrocytes were prepared as described earlier [50, 51]. In brief, primary astrocytes from T175 flasks (four flasks per one group) were cultured in DMEM with 10 % FBS. One day before the exosome preparation, culture medium was replaced to AIM-V medium w/ or without simvastatin. Culture supernatants of cells grown for 24 h in AIM-V medium were collected and spun at 300 g for 10 min to remove cells. The supernatants were then sequentially centrifuged at 1,200 g for 20 min, 10,000 g for 30 min, and 100,000 g for 1 h. The 100,000 g pellet was washed, and then again spun at 100,000 g for 1 h. The second 100,000 g pellet (exosomal pellet) was resuspended in PBS, and the supernatants were used as non-exosome fractions.

### Immunostaining

Immunocytochemical staining was performed as described previously [48, 52]. Briefly, the fixed cells were incubated with mouse anti-IDE (1:300) and/or anti-LC3B (1:300) primary antibodies in PBST (PBS with 0.2 % Triton X-100) buffer overnight at 4 °C. After several washes, the cells were incubated with secondary antibody, and images were taken using a confocal laser scanning microscope (FV10i-w; Olympus).

### Filipin staining

The filipin staining was determined as manufacturer's protocol (F9765, Sigma-Aldrich). Briefly, the cells were fixed with 4 % paraformaldehyde (PFA), and then were incubated with 25 μg/ml filipin in PBS for 30 min at room temperature. The images were taken using a confocal laser scanning microscope (FV10i-w; Olympus).

### Cell viability assay

To measure cell viability, we used MTS assay kit (#G3580, Promega, Madison, WI) according to the manufacturer’s instructions. In Brief, the cells in a 96-well microtiter plate were incubated in the absence or presence of various drugs. After 24 h incubation, we transferred an appropriate volume of MTS assay solution into 96-well plate and incubated the plate for 1 h at 37 °C in the dark. The absorbance was measured using a plate reader at 490 nm.

### Statistical analysis

For western blots, protein levels were normalized to pan forms or a housekeeping protein, such as β-actin or GAPDH. All data were expressed as means ± standard error of the mean (S.E.M.). Statistical analysis was performed using GraphPad Prism 5 (San Diego, CA, USA). The data were analyzed by one-way analysis of variance with *post-hoc* test or unpaired *t*-tested regarded as appropriate. *P* values of < 0.05 were considered statistically significant.

## Additional files

Additional file 1: Figure S1. Statins regulate cholesterol levels in astrocytes. (A) Cellular cholesterol levels were measured by filipin staining. MβCD is a positive control. (B) Quantitative analysis of Figure S1A using the Image J program (*N* = 3 experiments). ** *p* < 0.01, *** *p* < 0.001 vs. vehicle-treated cells. (PDF 217 kb) Additional file 2: Figure S2. Fluvastatin induces IDE secretion from astrocytes. (A) Increased IDE levels secreted from the primary astrocytes by fluvastatin in a concentration-dependent manner. Blots are representative of at least 3 independent experiments (*N* = 3 experiments). (B) Quantitative analysis of Figure S2A. ** *p* < 0.01 vs. vehicle-treated cells. (PDF 71 kb) Additional file 3: Figure S3. Statin-induced IDE secretion is mediated by both exosome- and non-exosome-dependent pathways. (A) Western blot analysis of IDE levels in the exosomes or non-exosome fractions. TSG101 is an exosome marker protein. (PDF 30 kb) Additional file 4: Figure S4. Cell death was not induced in this study. (A) MTS assay was used for checking cell viability under simvastatin, 3MA and/or bafilomycin treated condition. *N* = 5 experiments. (PDF 57 kb)

## Abbreviations

3-MA: 3-methyladenine
ACM: astrocyte-conditioned media
AD: Alzheimer’s disease
APP: amyloid beta precursor protein
Atg: autophagy-related
ATP: adenosine triphosphate
AVs: autophagic vacuoles
Aβ: amyloid beta
Baf: bafilomycin
BBB: blood-brain barrier
CaMKKβ: Ca^2+^/calmodulin-dependent protein kinase kinase-beta
DMEM: Dulbecco’s modified Eagle’s medium
ELISA: enzyme-linked immunosorbent assay
EM: electron microscopy
FBS: fetal bovine serum
GAPDH: glyceraldehyde 3-phosphate dehydrogenase
HMGB1: high-mobility Group Box 1
IDE: insulin-degrading enzyme
LC3: microtubule-associated protein 1A/1B-light chain 3
LKB1: liver kinase B1
MMP-9: matrix metalloproteinase-9
mTOR: mammalian target of rapamycin
PBS: phosphate buffer saline
PFA: paraformaldehyde
qPCR: quantitative real-time polymerase chain reaction
TCA: Trichloroacetic acid
WB: western blotting
