# Supplementary figures and images for: Statins induce insulin-degrading enzyme secretion from astrocytes via an autophagy-based unconventional secretory pathway
Source: Mol Neurodegener. 2015 Oct 31;10:56. doi: 10.1186/s13024-015-0054-3 (PMC4628355; doi:10.1186/s13024-015-0054-3)

Figure S1.

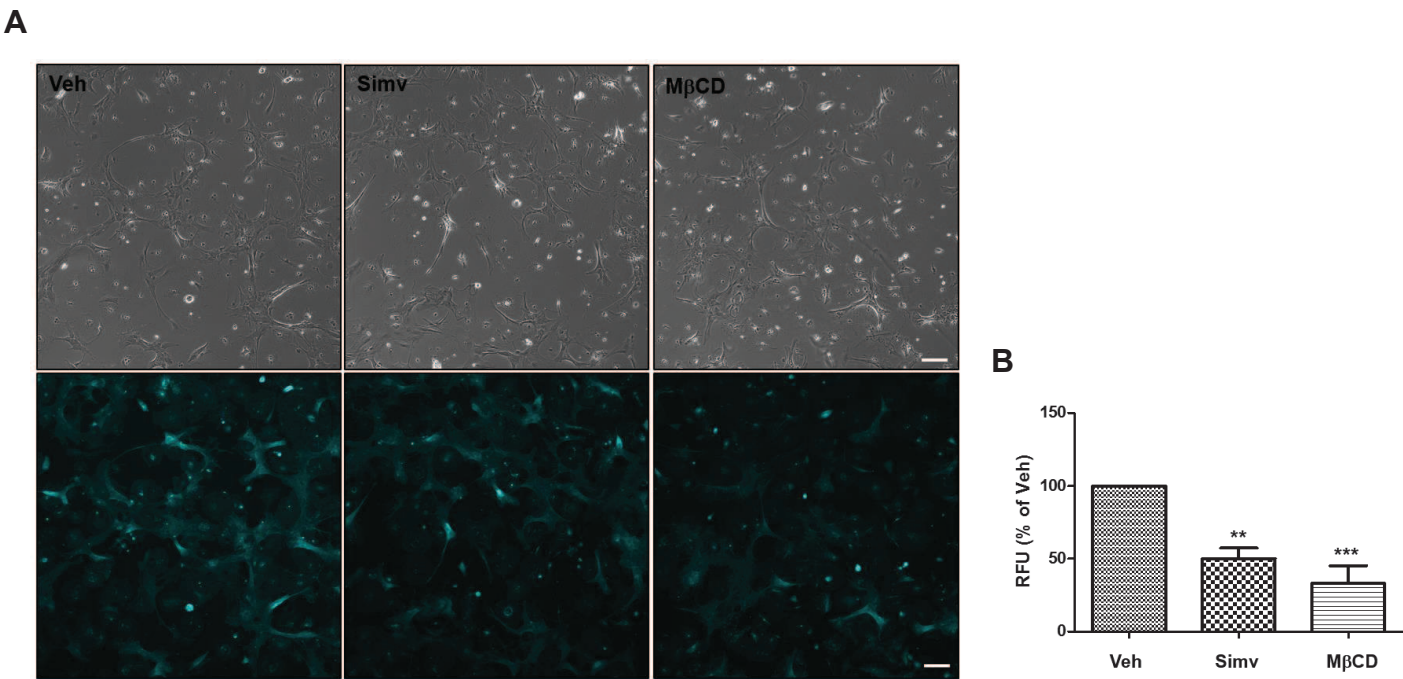

Supplement: Additional file 1: Figure S1. — Statins regulate cholesterol levels in astrocytes. (A) Cellular cholesterol levels were measured by filipin staining. MβCD is a positive control. (B) Quantitative analysis of Figure S1A using the Image J program (N = 3 experiments). ** p < 0.01, *** p < 0.001 vs. vehicle-treated cells. (PDF 217 kb) [file 13024_2015_54_MOESM1_ESM.pdf]

Figure S2.

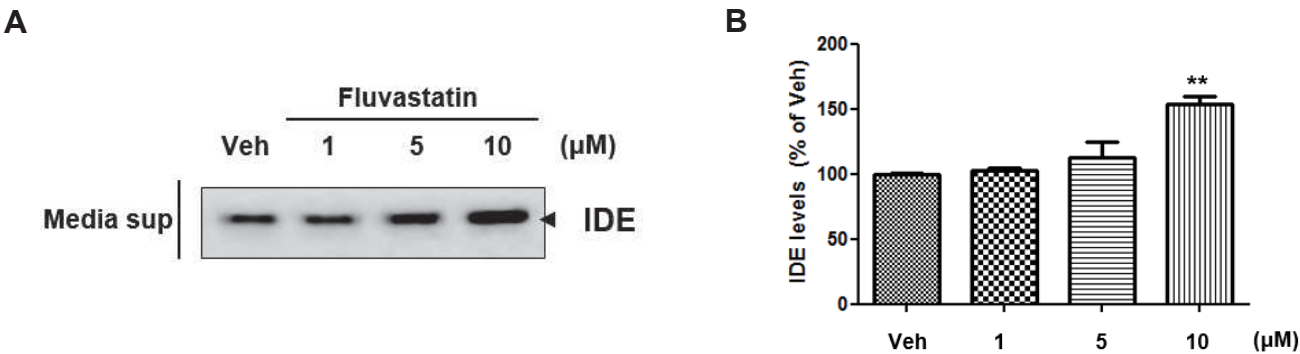

Supplement: Additional file 2: Figure S2. — Fluvastatin induces IDE secretion from astrocytes. (A) Increased IDE levels secreted from the primary astrocytes by fluvastatin in a concentration-dependent manner. Blots are representative of at least 3 independent experiments (N = 3 experiments). (B) Quantitative analysis of Figure S2A. ** p < 0.01 vs. vehicle-treated cells. (PDF 71 kb) [file 13024_2015_54_MOESM2_ESM.pdf]

Figure S3.

A

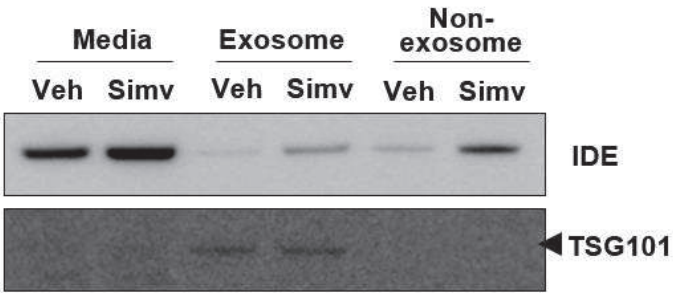

Supplement: Additional file 3: Figure S3. — Statin-induced IDE secretion is mediated by both exosome- and non-exosome-dependent pathways. (A) Western blot analysis of IDE levels in the exosomes or non-exosome fractions. TSG101 is an exosome marker protein. (PDF 30 kb) [file 13024_2015_54_MOESM3_ESM.pdf]

Figure S4.

A

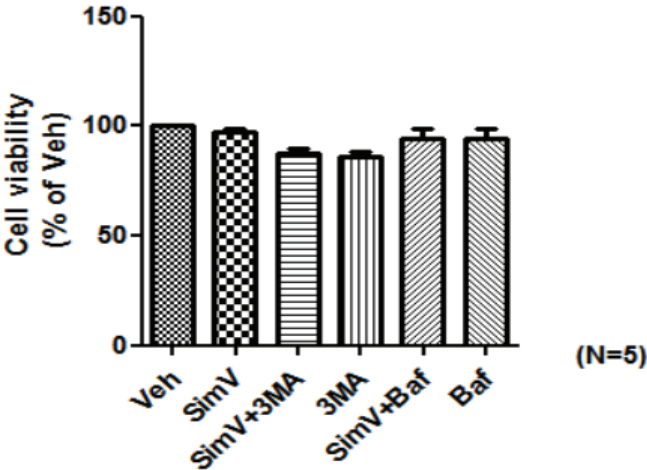

Supplement: Additional file 4: Figure S4. — Cell death was not induced in this study. (A) MTS assay was used for checking cell viability under simvastatin, 3MA and/or bafilomycin treated condition. N = 5 experiments. (PDF 57 kb) [file 13024_2015_54_MOESM4_ESM.pdf]
